# Trends in surgical stabilization of rib fractures: A contemporary literature review

**DOI:** 10.1016/j.jcot.2025.103028

**Published:** 2025-04-23

**Authors:** Matthew Masoudi, Ropafadzo Muchabaiwa, Elizabeth Wake, Bhavik Patel

**Affiliations:** a Trauma Service, Gold Coast University Hospital, Queensland, Australia; b School of Medicine, Griffith University, Gold Coast, Australia; c Bond University, Gold Coast Australia

**Keywords:** Rib fracture, Thoracic trauma, Surgical stabilization, Patient-reported outcome, Clinical outcomes

## Abstract

**Background:**

Blunt chest trauma leading to rib fractures is a common injury, accounting for 20 % of thoracic trauma cases. Surgical Stabilization of Rib Fractures (SSRF) has gained popularity due to advancements in surgical techniques and multidisciplinary care, resulting in improved patient outcomes. Despite a growing body of literature on SSRF, inconsistencies in study design and outcome reporting limit the synthesis of findings and the establishment of clear clinical guidelines. This scoping review aims to provide an overview of the existing SSRF literature, identifying prevalent trends and reported outcomes.

**Methods:**

A systematic scoping review was conducted following Arksey and O'Malley's framework and the Preferred Reporting Items for Scoping Reviews (PRISMA) guidelines. The study was registered with Open Science Framework (8V9KN). A comprehensive search of MEDLINE, EMBASE, CINAHL, and Cochrane CENTRAL was performed from inception to March 1, 2025. Studies were included if they reported on SSRF for blunt chest trauma in human patients. Data extraction focused on study characteristics, patient demographics, reported outcomes, and methodological rigor.

**Results:**

A total of 1462 articles were screened, with 185 studies meeting the inclusion criteria. The majority (N = 162, 88 %) were published between 2015 and 2025, with the highest number in 2022. Most studies (N = 144, 78 %) employed a cohort study design, predominantly retrospective (N = 115, 80 %), while randomized controlled trials (RCTs) constituted only 8 % (N = 15). Hospital outcomes, including length of stay (N = 112) and ICU stay (N = 97), were the most frequently reported measures. Complications were documented in 124 studies, with pneumonia (N = 90) and mortality (N = 94) being the most common. Patient-reported outcomes (PROMs) were included in 60 studies (32 %), with pain (N = 46, 78 %) and quality of life (N = 23, 39 %) as key measures. Device and procedural details were reported in 70 studies (38 %), with 62 using the same device. However, variations in outcome measurement and a predominance of retrospective designs limit comparability.

**Conclusion:**

SSRF literature has expanded significantly over the past decade, yet inconsistencies in study design and outcome reporting hinder the development of standardized clinical guidelines. Future research should prioritize prospective, multi-center trials with uniform reporting standards to enhance the reliability and applicability of findings.

## Graphical abstract

**Figure:**
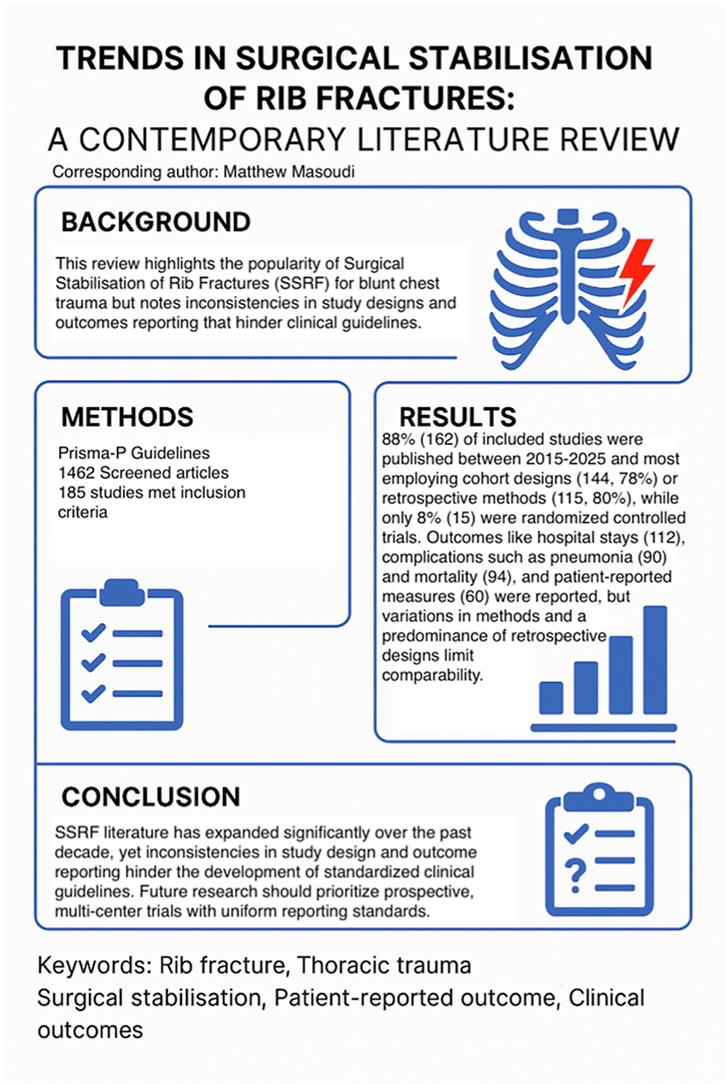

## 1 Background

Blunt chest trauma resulting in rib fractures is a common injury accounting for 20 % of patients who have suffered thoracic trauma.^1^ Traditionally, rib fractures causing unstable chest wall mechanics were one of the factors in predicting negative patient outcomes^2^ due to pulmonary complications.^3^, ^4^, ^5^, ^6^ Surgical Stabilization of Rib Fractures (SSRF) in the unstable chest wall has been incorporated into the management of thoracic trauma by surgical specialties and is increasingly gaining popularity.^7^ Advances in surgical fixation techniques, rib fixation hardware, and the delivery of care by a multi-disciplinary team approach have resulted in a reduction in both hospital and intensive care unit length of stays, and subsequent patient outcomes have resulted in increased support by clinicians for performing SSRF in this patient cohort.^8^

There is a plethora of literature about SSRF which is multifaceted and encompasses chest wall injury patterns best suited to SSRF, surgical procedure techniques, and organizational and patient-centred outcomes such as reduction in pain and time spent in hospital. Many studies that report positive outcomes following SSRF are single-center retrospective reviews, which can impact the quality and reproducibility of findings.^9^ When conservative management is compared with retrospective SSRF studies, conflicting reports are presented about longitudinal patient-reported outcome measures (PROMS).^10^^,^^11^ Several systematic reviews have also explored aspects of SSRF such as pulmonary function, complications, and multiple rib fracture patterns.^12^, ^13^, ^14^ While this improves the quality of the evidence produced, the breadth of topics discussed remains extensive, resulting in challenges when reviewing the literature due to the difficulty in consistency in the comparison of study outcomes, synthesizing findings, and establishing clear clinical guidelines.

The predominance of retrospective cohort studies in this area introduces biases such as selection bias and information bias, which impact the reliability of findings. To assist in identifying prevalent outcomes presented within the literature, an overview of the depth and breadth of the current SSRF evidence base is required. The primary aim of this review is to provide an overview of the current body of research on SSRF in order to understand the trends and quality of the evidence base. The secondary aim is to categorize and quantify the identified outcomes reported in the literature.

## 2 Methods

A systematic scoping review methodology was used as this is an approach that provides a framework to determine the current state of the available evidence on a specified topic.^15^ This methodology can be used to report on the extent, range, and nature of the available literature that informs practice. The framework by Arksey and O'Malley was used to guide the review, and the Preferred Reporting Items for Scoping Reviews statement for the reporting of scoping reviews was followed. The study was registered in Open Science Framework with the registration number 8V9KN.

### 2.1 Identifying relevant studies

To identify relevant studies for inclusion, the following electronic databases were searched: Medical Literature Analysis and Retrieval System Online (MEDLINE via OVID), EMBASE, Cumulative Index to Nursing and Allied Health Literature (CINAHL via OVID), and the Cochrane Central Register of Controlled Trials (CENTRAL; Cochrane Library). Databases were searched from inception to March 1st, 2025. Forward and backward citation searching of included articles was undertaken to identify additional literature. Duplicate publications were removed before the assessment of eligibility. The search strategy incorporated a combination of Medical Subject Headings (MeSH) terms and keywords related to “surgical stabilization of rib fractures,” “internal fixation of rib fractures,” “rib fracture fixation,” “flail chest surgery,” “thoracic trauma surgery,” and other related terms.

### 2.2 Study selection eligibility criteria

The authors selected all articles that met the following inclusion criteria: human (alive) patients, of any age, who have blunt chest wall trauma and who have undergone a surgical stabilization of rib fixation procedure. Articles were excluded for the following criteria: penetrating and non-traumatic chest-wall injury. Abstracts only, single-patient case studies, systematic reviews, guidelines, conference proceedings, and non-English articles were also excluded. All references were stored using a bibliographic citation manager. Eligible articles were selected through two phases using Rayyan. In phase one, the reviewers (MM, RM) independently reviewed the titles and abstracts of the articles that met the inclusion criteria. In the second phase, the full texts of the remaining articles were reviewed following the same process. Disagreements about study inclusion were discussed and resolved through consensus, with the final decision completed by a third author (BP).

### 2.3 Charting the data

For the articles that were included following the screening process, data relating to demographics of the article (country of origin, publication year), and the reported pre-identified outcome categories of hospital outcomes, complications, patient-reported outcomes, and procedural and device-related aspects were extracted. Data extraction was completed by two members of the review team (MM, RM). The content of the database was developed and refined as data were extracted.

Collating, summarizing, and reporting the results Descriptive statistics are reported in frequency and percentages. All articles were assessed for methodological rigor using the CASP Quality checklist.^16^ All studies were included regardless of quality.

## 3 Results

### 3.1 Search outcome

**Fig. 1:**
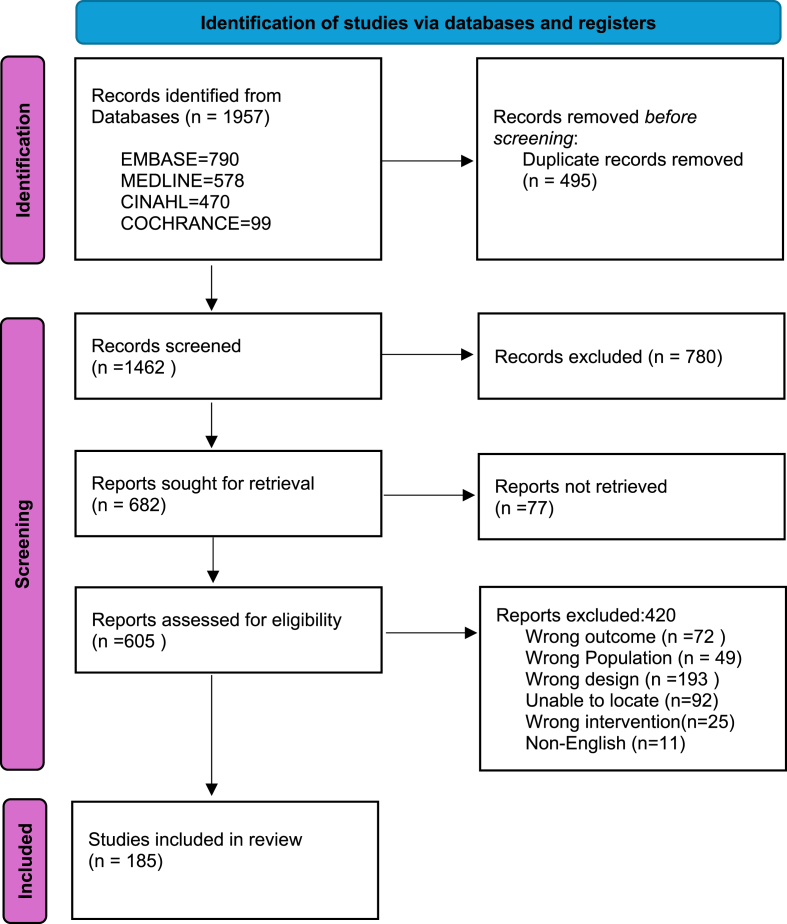
Prisma Flow Chart. *From:* Page MJ, McKenzie JE, Bossuyt PM, Boutron I, Hoffmann TC, Mulrow CD et al. The PRISMA 2020 statement: an updated guideline for reporting systematic reviews. BMJ 2021; 372:n71. doi: 10.1136/bmj.n71.

**Fig. 2:**
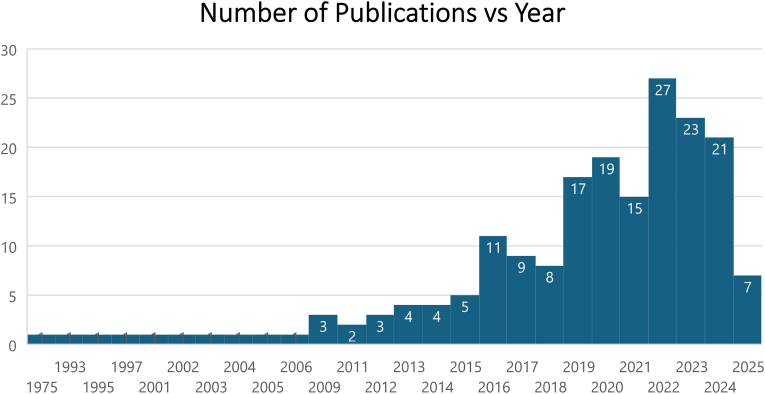
Number of publications by year. Data for 2025 includes studies published until March 1st 2025.

A total of 1462 articles were screened with 780 records excluded due to being duplicates, non-English, and wrong design. After screening four hundred and twenty were excluded (Fig. 1). One hundred and eighty-five articles were included in the full-text review and data was extracted (see Fig. 2).

### 3.2 Publication summary

Publication years ranged from 1975 to 2025, with the majority (N = 162, 88 %) published in the last decade (2015–2025). The highest number of publications occurred in 2022, and publication rates have remained consistently high since. Notably, data for 2025 includes only studies published up to March 1.

Studies were conducted in 18 countries the majority of which were from the United States of America (USA) (N = 77, 42 %). Australia, China, and the United Kingdom (UK) collectively accounted for 26 % (N = 48) of studies, with 16 publications included from each country.

Over three-quarters of the studies (n = 144, 78 %) used a cohort study design, of which 80 % (N = 115) were retrospective. Randomized controlled trials (RCT) accounted for only 8 % of the included studies (n = 15). Thirty-eight (20 %) studies were multi-centred. Nine studies were sponsor-driven (i.e. the makers of rib fixation devices), which were a mixture of designs including cohort (n = 6), RCT (n = 3), and a single case series.

### 3.3 Patient population

Studies included patients affected by multiple injuries (n = 120, 65 %) which included the chest and isolated chest injuries (n = 71, 38 %). Almost three-quarters of studies (n = 139, 75 %) excluded patients who had sustained a traumatic brain injury in addition to their blunt chest trauma.

An Injury Severity Score (ISS), which depicts a patient's injury severity in relation to mortality (Marasco et al., 2019) was identified in 139 articles (75 %); of those that reported the ISS, the median ISS was 21 (IQR 18–25). Mechanism of injury was reported in 92 studies (50 %), with motor vehicle accidents the most commonly reported mechanism of injury (n = 84, 92 %). Falls were reported in 25 studies (30 %) and crush injuries, assault, and CPR were reported in one study respectively.(see Fig. 3)

### 3.4 Reported categories

**Fig. 3:**
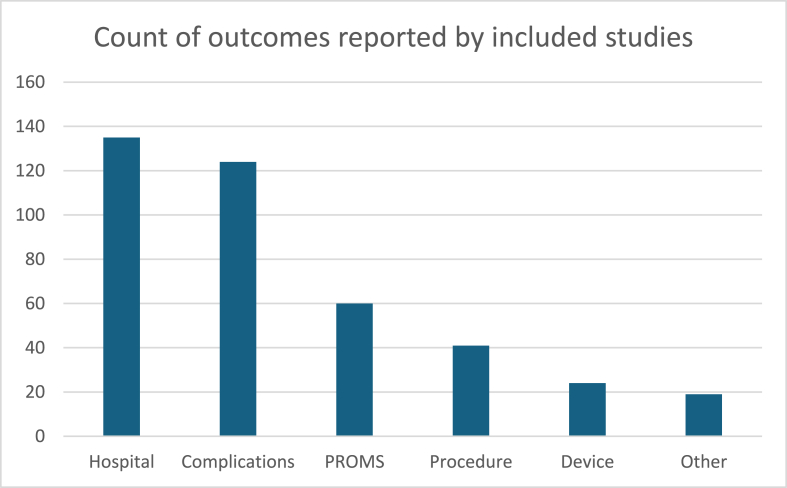
Reported outcomes.

### 3.5 Hospital outcomes

Hospital outcomes were the most frequently reported outcome (n = 135). The length of hospital stay was frequently reported (n = 112) followed by the overall length of intensive care unit stay (n = 97). Mechanical ventilation duration was reported in 90 studies.

Time to operative intervention was documented in 32 studies, all of which were published from 2013 onwards. Fifteen studies (8 %) incorporated a health economic analysis; of which, all but four were published after 2017 (n = 9).

### 3.6 Complications

One hundred twenty-four studies reported complications, with mortality (n = 94) and pneumonia (n = 90) being the most commonly documented. Extra-pulmonary infections were reported in 28 studies (22 %), while 19 studies (15 %) mentioned sepsis; however, none provided a definition or criteria for diagnosing sepsis. The cause and timing of death were detailed in 10 studies (8 %).

Twenty studies specified the timeframe for complications, indicating the number of days post-admission when they were observed. While 50 studies included a follow-up component for complications, none clarified whether these complications occurred during hospitalization or the follow-up period, limiting the ability to assess long-term risk.

### 3.7 Patient reported outcomes

PROMs were collected in 60 studies (32 %); over half (n = 27, 56 %) were published from 2017 onwards. Pain (n = 46, 78 %) and health-related quality of life (HRQOL) (n = 23, 39 %) were commonly measured with assessment tools including EQ-5D-3 and 5L, Short Form 12 and 36, and verbal rating scales. Follow-up was reported in fifty-five studies (30 %) and ranged from 2 to 24 months. The collection of PROMs were reported in 34 studies (61 %) which reported a follow-up.

### 3.8 Device and procedure

Seventy studies (38 %) reported details of the surgical procedure, with 27 (39 %) specifically documenting the materials and/or devices used. Of these, 62 studies used the same device, while the remaining studies reported the use of nine different devices. Four of the studies that identified specific devices were sponsor-driven. Additionally, 13 studies (8 %) examined the health economics of the devices and materials used in the procedure.

## 4 Discussion

The aim of this scoping review was to provide an overview of the current SSRF literature and to quantify consistently reported outcomes. Outcomes in relation to hospital admissions such as length of stay and complications were frequently reported; less than half of the studies reported patient-reported outcomes such as pain and quality of life. Additionally, whilst complications were frequently documented, it is unclear how these were measured, which poses a challenge when drawing comparisons.

The multifactorial differences in the setup of trauma systems around the globe is a well-known fact.^14^^,^^17^ This may lead to differences in the outcomes measurement from a surgical procedure. Given the geographical disparities and differences in trauma systems across the globe, outcomes should be measured and reported consistently to ensure relevance and applicability. However, for SSRF, such consistency may not be possible due to the dominance of literature from the USA, which may lead to potential discrepancies and misinterpretation of results.

Trauma care structures vary significantly worldwide, influencing SSRF outcomes and study findings. For example, in the USA, Level 1 trauma centers often have dedicated thoracic surgeons and standardized SSRF protocols, while in other regions, rib fixation may be performed by general surgeons with variable expertise.^18^, ^19^, ^20^ Similarly, access to post-discharge rehabilitation services varies, affecting long-term patient-reported outcomes. These disparities underscore the need for region-specific guidelines while promoting globally consistent reporting standards to enhance the applicability of research findings to diverse healthcare settings.

Organizations such as the Chest Wall Injury Society (CWIS), the Royal Australasian College of Surgeons (RACS), and the American College of Surgeons (ACS) have a pivotal role in providing expectations and guidelines on both the nature of data to be reported and how to measure it effectively. A unified and coherent code provided by these organizations would greatly assist in promotion of the uniformity and interpretation of SSRF literature across different healthcare systems.

### 4.1 Quality of evidence

The majority (80 %) of included studies were retrospective cohort studies, a design prone to biases such as selection bias and information bias. While the CASP quality checklist was used to assess study quality, the methodological robustness of these studies varied. Some studies suffered from small sample sizes, lack of control groups, and incomplete follow-up data, potentially affecting the generalizability of the findings. Lower-quality studies may have disproportionately influenced the review results, particularly in hospital outcome measures such as length of stay and complication rates, where patient selection criteria and data collection methods varied widely. To mitigate these issues, future SSRF research should prioritize prospective, multi-center trials with standardized outcome reporting to enhance the reliability of conclusions.

Retrospective cohort studies are inherently susceptible to biases that can impact the interpretation of hospital metrics such as length of stay and complication rates. Selection bias is particularly significant, as patients undergoing SSRF may systematically differ from those managed non-operatively. Multiple confounding factors, including comorbidities, trauma severity, and associated injuries, further influence SSRF outcomes. While the Injury Severity Score (ISS) was reported in 71 % of studies, variations in patient populations hinder direct comparisons. The lack of standardized control groups further complicates cross-study comparisons. Additionally, information bias remains a concern, as retrospective data collection may be incomplete or inconsistently recorded.

Standardizing outcomes, for surgical stabilization of rib fractures, could enhance the ability to compare different studies, increasing the reliability and validity of the conclusions drawn from these comparisons. Through the standardization of outcomes, including in the context of single-center studies, results become valuable for meta-analysis and systematic reviews, providing a more comprehensive view of the evidence. The absence of standardized outcomes in SSRF studies may contribute to the observed differences between retrospective and prospective studies. Without standardized outcomes, the results from both types of studies can be challenging to compare due to the different measures of success, leading to conflicting conclusions about the effectiveness of SSRF.

Ingoe et al., conducted a multi-stakeholder Delphi consensus study to establish a standardized framework for assessing outcomes in clinical trials related to surgical fixation of rib fractures.^21^ Identified outcomes included adverse events, mortality, quality of life, and functional parameters. However, consensus on a standardized follow-up framework was not reached, highlighting the ongoing challenge of achieving uniformity in outcome reporting in SSRF research.

Among the 185 included studies, 50 incorporated patient follow-ups in their research design, yet the methods and lengths of follow-up varied, compromising the consistency and comparability of results. Standardizing follow-up procedures is key to ensuring a thorough and uniform assessment of SSRF outcomes.^21^ Further research aimed to establish a uniform follow-up framework that could enable a comprehensive evaluation and offer a better view of SSRF's efficacy and impact.

### 4.2 Patient reported outcome measures

The rising importance of patient-reported outcome measures in healthcare research is a reflection of the increasing emphasis on patient-centric care and consumer engagement. These measures, which capture patients' perceptions of their health status and treatment outcomes, are becoming prevalent in research due to a shift towards shared decision-making and personalized medicine.^22^^,^^23^

The use of PROMs in healthcare has seen significant growth,^24^ with some countries mandating their use for specific elective surgical procedures.^25^ This approach allows for a better understanding of both short- and long-term recovery patterns, helping to identify predictors of outcomes.^26^ However, this review shows that literature on SSRF continues to focus on metrics such as hospital stay, mortality rate, cost savings and procedural effectiveness, which may overshadow the importance of health outcomes that matter to patients, such pain and quality of life. This perspective has been highlighted by patient groups who prioritize these outcomes over cost-related factors.^21^^,^^27^

This review highlights inconsistencies in PROM measurement, an area that requires attention. Standardized PROMs such as the EQ-5D, SF-36, and Visual Analog Scale for pain could be adopted to ensure comparability across studies. Organizations like CWIS and ACS could play a pivotal role in developing and endorsing a core outcome set for SSRF research. However, challenges in harmonization persist, particularly due to differences in healthcare systems, resource availability, and patient follow-up capabilities across countries. A potential solution is the implementation of international consensus guidelines that mandate specific PROM tools and follow-up durations for SSRF studies.

## 5 Conclusion

The surge in SSRF literature over the last decade reflects a heightened clinical interest in managing chest wall injuries. Positive outcomes in small randomized controlled trials and advancements in techniques may have contributed to this trend.^17^^,^^18^ In terms of translation towards knowledge, caution is warranted due to the predominance of retrospective, single-center studies, as this might be secondary to ambiguity about the efficacy of SSRF as expressed by clinicians.^19^ This highlights the need for multi-center studies with standardized reporting protocols to improve the quality of evidence and consistency around all trauma centers.

## 6 Limitations

The review was limited by including only articles published in English. Furthermore, only prominent databases were searched, which could result in missed studies.

## CRediT authorship contribution statement

**Matthew Masoudi:** conducted the literature search for this study while, wrote the background, the methods, wrote the results and discussion sections. **Ropafadzo Muchabaiwa:** conducted the literature search for this study while, wrote the results and discussion sections. **Elizabeth Wake:** conducted the literature search for this study while, the methods. **Bhavik Patel:** provided expert consultation, wrote the background, All authors reviewed and edited the entire document to ensure its accuracy and completeness.

## Guardian/patient's consent statement

Not required, Not applicable.

## Ethical statement

Not required, Not applicable.

## Source of funding

None.

## Declaration of competing interest

The authors declare that they have no known competing financial interests or personal relationships that could have appeared to influence the work reported in this paper.
